# A DYADIC ASSESSMENT OF COGNITIVE RESOURCES AND COGNITIVE FUNCTION AMONG OLDER KOREAN AMERICAN COUPLES

**DOI:** 10.1093/geroni/igad104.2617

**Published:** 2023-12-21

**Authors:** Nan Park, Yuri Jang, Kyungmin Kim, Soondool Chung, William Haley, David Chiriboga, Miyong Kim

**Affiliations:** University of South Florida, Tampa, Florida, United States; University of Southern California, Los Angeles, California, United States; Seoul National University, Seoul, Republic of Korea; Ewha Womans University, Seoul, Republic of Korea; University of South Florida, Tampa, Florida, United States; University of South Florida, Tampa, Florida, United States; University of Texas at Austin, Austin, Texas, United States

## Abstract

Individuals’ health and well-being are often shaped by significant others in their lives in that spousal relationships are particularly important. In dyadic studies among older couples, few studies have examined cognitive health as an outcome variable. Considering the lack of couple research on cognition especially among older immigrant population, the purpose of this study was to examine the cognitive function of community-dwelling older Korean American couples in the U.S. Data were drawn from a survey with older Korean Americans aged 60 and older, collected during 2017−2018 in multiple locations. Using a subset of 252 couples of the survey (individual N = 504), we examined the extent to which one’s cognitive function was associated with the cognitive resources (education, acculturation, friend network, and activity participation) of their own and of their spouse. The analyses with the Actor-Partner Interdependence Model showed the differences in the actor- and partner-effects between spouses. For husbands, acculturation was the only significant actor-level predictor to cognitive function. Wives’ cognitive function was associated with a wider array of their own cognitive resources, including education, friend network, and activity participation. The partner effect was only observed in education for husbands, indicating that wives’ higher education was beneficial for their husbands’ cognitive function. Further, the cognitive benefit of higher education was found to be maximized for husbands who also had greater levels of acculturation or activity participation. For practice implications, cognitive resources both in individual and spousal levels could help promote the cognitive health and well-being of older married couples.

